# Subtypes of Ovarian Cancer and Ovarian Cancer Screening

**DOI:** 10.3390/diagnostics7010012

**Published:** 2017-03-02

**Authors:** Masafumi Koshiyama, Noriomi Matsumura, Ikuo Konishi

**Affiliations:** 1 Department of Gynecology and Obstetrics, Kyoto University, Graduate School of Medicine, Sakyo-ku, Kyoto 606-8507, Japan; noriomi@kuhp.kyoto-u.ac.jp (N.M.); konishi@kuhp.kyoto-u.ac.jp (I.K.); 2 Department of Women’s Health, Graduate School of Human Nursing, The University of Shiga Prefecture, 2500 Hassakacho, Hikone, Shiga 522-8533, Japan; 3 Department of Obstetrics and Gynecology, National Hospital Organization Kyoto Medical Center, Fushimi-ku, Kyoto 612-8555, Japan

**Keywords:** subtypes, two types of ovarian cancer, ovarian cancer screening, CA125, transvaginal sonography

## Abstract

Ovarian cancer is the foremost cause of gynecological cancer death in the developed world, as it is usually diagnosed at an advanced stage. In this paper we discuss current issues, the efficacy and problems associated with ovarian cancer screening, and compare the characteristics of ovarian cancer subtypes. There are two types of ovarian cancer: Type I carcinomas, which are slow-growing, indolent neoplasms thought to arise from a precursor lesion, which are relatively common in Asia; and Type II carcinomas, which are clinically aggressive neoplasms that can develop de novo from serous tubal intraepithelial carcinomas (STIC) and/or ovarian surface epithelium and are common in Europe and the USA. One of the most famous studies on the subject reported that annual screening using CA125/transvaginal sonography (TVS) did not reduce the ovarian cancer mortality rate in the USA. In contrast, a recent study in the UK showed an overall average mortality reduction of 20% in the screening group. Another two studies further reported that the screening was associated with decreased stage at detection. Theoretically, annual screening using CA125/TVS could easily detect precursor lesions and could be more effective in Asia than in Europe and the USA. The detection of Type II ovarian carcinoma at an early stage remains an unresolved issue. The resolving power of CA125 or TVS screening alone is unlikely to be successful at resolving STICs. Biomarkers for the early detection of Type II carcinomas such as STICs need to be developed.

## 1. Introduction

Ovarian cancer is the foremost cause of gynecological cancer death and is overall one of the most frequent causes of fatal malignancy in women [1]. The symptoms are often nonspecific, hampering early detection, so the majority of patients present with advanced-stage disease.

Screening is defined as the application of a test or a combination of tests to an asymptomatic at-risk population to detect a disease at an earlier and more curable stage. In 2011, an examination of a screening program for prostate, lung, colorectal, and ovarian cancer (PLCO) in the USA revealed that annual screening using CA125/transvaginal sonography (TVS) did not markedly reduce the ovarian cancer mortality rate [2,3]. While this finding suggests that it is not possible to detect ovarian cancer at an earlier curable stage, it is possible to question the validity of these data.

Recently, the characteristics of several subtypes of ovarian cancer have been elucidated by the findings from histopathological, molecular, and genetic studies. Ovarian cancer can be roughly divided into two broad categories: Type I, in which precursor lesions in the ovaries have clearly been described; and Type II, in which such lesions have not been clearly described and tumors may develop de novo from the tubal and/or ovarian surface epithelium [4]. Understanding these characteristics is important in the effort to reduce ovarian cancer mortality.

This study first describes the characteristics of the subtypes of ovarian cancer and the results of several large-scale studies of ovarian cancer screening. We discuss current issues, the efficacy and problems associated with ovarian cancer screening, and make comparisons of the characteristics of ovarian cancer subtypes.

## 2. Ovarian Carcinoma Types

### 2.1. Type I Carcinoma

Type I carcinomas are generally slow-growing indolent neoplasms, and their precursor lesions in the ovaries have been clearly described [4].

#### 2.1.1. Endometrioid Carcinoma and Clear Cell Carcinoma

Clear cell and endometrioid carcinomas are believed to arise from endometriosis of the ovary. Among malignant transformation cases of endometriotic cyst, serial transvaginal ultrasonography (USG) examinations revealed an increase in the cyst size [5]. Increased risks of ovarian carcinoma arising from endometriosis were associated with infertility, early menarche, and late menopause [6]. Pathologically, the co-existence of ovarian carcinoma and endometriosis is frequently observed, and in such cases endometriosis is called “atypical endometriosis”, a putative precursor lesion including atypia of the cell nucleus [7].

Carcinogenesis of endometrioid and clear cell carcinomas arising from endometriotic cysts is significantly influenced by the microenvironment in the precursors [8]. The content of an endometriotic cyst (including free iron in old blood) is thought to be associated with cancer development through the induction of persistent oxidative stress [9]. The epithelial cells in the cyst are exposed to oxidative stress and hypoxia. Thus, they are subject to increased cellular and DNA damage, have less efficient DNA repair, and are easily transformed [10,11].

Somatic mutations in the ARID1A tumor-suppressor gene have been frequently identified in clear cell carcinoma. BAF250a encoded by ARID1A is a member of the SWItch/sucrose nonfermentable (SWI/SNF) complex. We recently reported that clear cell carcinomas exhibiting the loss of one or multiple SWI/SNF complex subunits demonstrated aggressive behaviors and poor prognosis [12].

#### 2.1.2. Mucinous Carcinoma

A subset of mucinous carcinomas is thought to develop in association with ovarian benign teratomas; however, the majority of mucinous carcinomas do not show any teratomatous components [13,14]. Other theories of an ontogeny include origin from mucinous metaplasia of surface epithelial inclusions, endometriosis, and Brenner tumors [5,14]; however, these observations are relatively uncommon, except for Müllerian endocervical mucinous or mixed borderline tumors [15,16].

Morphological transitions from cystadenoma to a mucinous borderline tumor (MBT) to intraepithelial carcinoma and invasive carcinoma have occasionally been observed [17]. An increasing frequency of KRAS mutations at codons 12 and 13 has been reported in cystadenomas, MBTs, and mucinous carcinomas [18,19,20,21]. These findings support the hypothesis of the “mucinous adenoma–carcinoma sequence” [17,22] and the view that mucinous carcinomas may develop in a step-wise fashion from mucinous cystadenomas and MBTs.

#### 2.1.3. Low-Grade Serous Carcinoma

Low-grade serous carcinomas are very rare tumors. They are genetically stable and are characterized by their low number of genetic mutations; therefore, they develop slowly from the precursors and behave in an indolent fashion. They are also thought to grow in a step-wise fashion from benign serous cystadenoma to serous borderline tumors (SBTs), and then to low-grade serous carcinoma.

p53 mutations are uncommon in low-grade serous carcinoma [23]. These carcinomas have a DNA content and level of copy number alterations that closely resembles that of SBTs [24,25].

One theory of the origin of these tumors is that they are derived from ovarian epithelial inclusions that have undergone Müllerian metaplasia [26]. The exposure of the mesothelial cells to the ovarian stromal microenvironment may result in transformation to Müllerian epithelium.

Another theory is that serous tumors may be derived from a secondary Müllerian system, arising from the embryological remnants of the proximal Müllerian ducts located within the ovarian hilm [27,28]. However, a new theory suggests that low-grade serous carcinoma may be derived from the fallopian tube. The premise is that shed tubal epithelial cells can implant on the ovarian surface epithelium, followed by the formation of inclusion cysts and transforming serous carcinoma [29,30].

### 2.2. Type II Carcinoma

Type II carcinomas are clinically aggressive neoplasms and may develop de novo from the tubal and/or ovarian surface epithelium.

#### High-Grade Serous Carcinoma

High-grade serous carcinomas account for 68% of ovarian cancer and have the worst prognosis, as they are high-grade clinically aggressive neoplasms that are usually diagnosed at an advanced stage. They show TP53 gene mutations in nearly 80% of cases [31,32,33,34] and have a high Ki67 proliferation index (50%–75%). Chromosomal rearrangements are common and associated with gene instability. Mutations in the *BRCA* 1 and 2 genes are associated with 90% of hereditary high-grade serous carcinoma cases [35].

Recently, analyses of gene expression microarray data from The Cancer Genome Atlas (TCGA) project have revealed that high-grade serous carcinoma can be classified into one of four gene expression subtypes: mesenchymal, immunoreactive, proliferative, and differentiated [36,37]. Our group reported that the progression-free and overall survival were best in the immunoreactive group, whereas the overall survival was worst in the mesenchymal transition group (*p* < 0.001 for each) [38]. Expression of vascular endothelial growth factor (VEGF) inhibits tumor immunity through the accumulation of myeloid-derived suppressor cells, and contributes to poor prognosis [39].

These tumors may develop de novo from the tubal and/or ovarian surface epithelium. In 2001, Piek et al. [40] found new transformations from hyperplastic to dysplastic lesions on tubal segments removed from women who had either *BRCA* mutations or a strong family history of ovarian carcinoma and underwent a risk-reducing bilateral salpingo-oophorectomy (BSO). These dysplastic lesions within the tubal epithelium are termed “serous tubal intraepithelial carcinomas” (STIC) and microscopic disease.

A very early abnormality termed “secretory cell outgrowths” (SCOUTs) was recently reported in tubal epithelia [41]. The TP53 signatures were the next earliest entities, and have an immunohistochemical definition of “p53-positive with a low proliferative index (Ki67 < 10%)”. Developing later were “serous tubal intraepithelial lesions” (STILs) [42], also known as “transitional intraepithelial lesions of the tube” (TILTs) by some authors. These have proliferative p53 signatures, tubal dysplasia, and even tubal epithelial atypia [40,43]. Lastly, these turned into STICs; thus, STICs appear to be associated with the development of serous carcinoma.

It was recently reported that the junction of the fallopian tube epithelium with the mesothelium of the tubal serosa might be a potential site for carcinogenesis [44]. Carcinomas arising from this junctional zone can easily invade the extensive lymphovascular system under the tubal epithelium and rapidly spread throughout the abdominal cavity.

In contrast, ovarian hilum cells have shown increased transformation potential after the inactivation of tumor suppressor genes transformation-related protein 53 (Trp53) and retinoblastoma 1 (Rb1) in mice [45]. These stem cells may also be the origin of high-grade serous carcinoma.

## 3. Large-Scale Studies of Ovarian Cancer Screening

Ovarian cancer screening was once thought to be ineffective, but has recently been reported to result in a better prognosis than without screening [46].

### 3.1. A Screening Program for Prostate, Lung, Colorectal, and Ovarian Cancer

One large-scale study of ovarian cancer screening examined a screening program for prostate, lung, colorectal, and ovarian cancer (PLCO) in the USA, performed using a randomized controlled trial (RCT) [2,3]. The annual screening in this study was performed by transvaginal sonography and CA125 level measurements.

The PLCO screening arm involved 78,216 women receiving either annual screening (*n* = 39,105) or the usual care (*n* = 39,111). Ovarian cancer was diagnosed in 212 patients (0.54%) in the screening group and 176 patients (0.45%) in the standard care group. The stage distribution in the screening group was as follows: 32 (15%) cases of Stage I disease, 15 (7%) cases of Stage II disease, 120 (57%) cases of Stage III disease, and 43 (20%) cases of Stage IV disease, indicating that 77% of patients had cancer at Stage III or higher. The distribution of cancer histologies included 116 (80%) cases of serous carcinomas, five (3%) cases of mucinous carcinomas, 19 (13%) cases of endometrioid carcinomas, and six (4%) cases of clear cell carcinomas, indicating that most cases involved serous cancers.

The authors concluded that annual screening did not reduce the ovarian cancer mortality rate compared with standard care. Based on this report, ovarian cancer screening is not considered to be effective.

### 3.2. Re-Analysis of the PLCO Screening Data

We obtained the authors’ datasets and performed a new analysis. We divided the patients who were diagnosed with ovarian cancer into two groups. One group included 101 patients whose ovarian cancers were detected through annual screening (CA125 and/or TVS) or within one year after screening. The other group included 344 patients in the screening group whose ovarian cancers were found at more than one year after screening due to the patient experiencing symptoms, as well as patients in the no screening and control groups. We previously reported these results [47]. The prognosis was significantly better in the patients in the former group than in those in the latter group (median survival: 6.1 vs. 3.3 years, *p* = 0.0017). Additionally, the first group contained significantly fewer Stage IV cases than the second group (13% vs. 29%, respectively, *p* = 0.005).

We identified two weaknesses in the PLCO screening: the group undergoing annual screening included many women who never received screening, and many patients with ovarian cancer in the screening group were diagnosed incidentally more than one year after screening, and as such could not be related to the direct effect of screening.

### 3.3. The United Kingdom Collaborative Trial of Ovarian Cancer Screening

The United Kingdom Collaborative Trial of Ovarian Cancer Screening (UKCTOCS) is an RCT of 202,638 women (control: 101,359; multimodal screening (MMS): 50,640; TVS alone: 50,639) [48,49,50]. The MMS protocol included annual CA125 screening interpreted using a patented “Risk of Ovarian Cancer” algorithm (ROCA) with TVS as a second-line test [51,52]. Ovarian cancer was diagnosed in 38 (0.08%) patients in the MMS group and 32 (0.06%) patients in the TVS group. The distribution of the cancer histologies was similar to that of the PLCO group. The distribution of the cancer stages in the MMS group was as follows: 17 (45%) patients with Stage I disease, 2 (5%) patients with Stage II disease, 19 (50%) patients with Stage III disease, and 0 (0%) patients with Stage IV disease, which was similar to that of the TVS group. Recently, a UK team reported on the final mortality, citing an overall average mortality reduction of 20%, and a reduction of 8% in years 0–7 and 28% in years 7–14 in the MMS group, compared with the no screening group [46]. They suggested that this late effect of screening was predictable given the unavoidable time interval from randomization to diagnosis and finally death. Therefore, their interpretation was that MMS screening was more effective after seven years of screening.

Very recently, Pavik pointed out two problems raised by the work of the UKCTOCS [53]. The UKCTOCS results from the analysis using the Cox proportional hazards model and the Royston–Parmar flexible parametric model indicated only small differences between the MMS and TVS modalities that were not statistically significant (estimated mortality reduction for years 7–14: 23% MMS vs. 21% TVS with the Royston–Parmar flexible parametric model). Another problem was that an expected lack of CA125 expression (20%) produces CA125-negative ovarian carcinomas that cannot be expected to be detected in the MMS group.

### 3.4. The Kentucky Screening Study

In the Kentucky Screening Study, single-arm annual TVS screenings of 37,293 women was performed [54,55]. The stage distribution of the 47 invasive ovarian cancers was as follows: 22 (47%) Stage I lesions, 11 (23%) Stage II lesions, 14 (30%) Stage III lesions, and 0 (0%) Stage IV lesions, with a 70% rate of Early-Stage (I/II) disease. The distribution of cancer histologies included 38% with serous carcinomas, 2% with mucinous carcinomas, 26% with endometrioid carcinomas, 4% with clear cell carcinomas, and 30% with others. The survival rate at five years of the patients with ovarian cancer in the annual screening group was better than that of the patients with ovarian cancer who did not undergo screening (74.8% ± 6.6% vs. 53.7% ± 2.3%, *p* < 0.001). Histologically, compared with the PLCO data, the rate of serous carcinomas was relatively low and the rate of endometrioid carcinomas was relatively high.

The authors concluded that annual TVS screening was associated with a decreased stage at detection, as well as a decrease in the case-specific ovarian cancer mortality. However, this study was not an RCT.

### 3.5. The Japanese Study

In Japan, the results of the Shizuoka Cohort Study of Ovarian Cancer Screening have been reported [56]. This study was an RCT of 82,487 low-risk postmenopausal women (intervention group: 41,688, control group: 40,799) who were screened using annual TVS and CA125 levels. The total number of cases of ovarian cancer in the screening group was 27 (0.06%). The stage distribution in the intervention group was as follows: 17 (63%) cases of Stage I disease, 1 (4%) case of Stage II disease, 7 (26%) cases of Stage III disease, and 2 (7%) cases of Stage IV disease. The distribution of the cancer histologies included 8 (30%) cases of serous carcinomas, 4 (15%) cases of mucinous carcinomas, 5 (19%) cases of endometrioid carcinomas, 9 (33%) cases of clear cell carcinomas, and 1 (4%) case of “other”. Histologically, most of these cases involved cancers other than serous carcinoma. The proportion of Stage I/II ovarian cancers was higher in the screening group (67%) than in the control group (44%). The rate of complete surgical excision was higher in the screening group (21; 78%) than in the control group (15; 47%) (*p* = 0.018). However, the mortality rates are unknown, which again is problematic.

## 4. Differing Histological Subtypes of Ovarian Carcinoma among Races

In Europe, the USA, and Asia, there are significant differences in the rates of histological subtypes of ovarian carcinoma [57,58,59,60,61,62]. As we reported previously, the rate of aggressive ovarian cancer such as high-grade serous cancer (Type II) is significantly higher in Europe and the USA than in Asia (*p* < 0.001) [47]. For example, the rates of Type I vs. Type II are, 24% vs. 48% in Europe (including the UK); 24% vs. 66% in Denmark; and 30% vs. 45% in the USA. Conversely, Type I carcinomas—indolent carcinomas arising from precursors—are relatively common in Asia. For example, the rates of Type I vs. Type II are 53% vs. 33% in Japan; 58% vs. 24% in Hong Kong; and 66% vs. 34% in Korea. These results theoretically imply that ovarian cancer screening using CA125/TVS would be more effective in Asia than in Europe and the USA, as the precursors or ovarian cancer can be detected at an earlier stage, thereby reducing the mortality.

## 5. Conclusions

We presented characteristics of subtypes of ovarian cancer, summarized in Table 1. Type I carcinomas are generally slow-growing indolent neoplasms, and their precursor lesions in the ovaries have clearly been described and are easily detected. Conversely, Type II carcinomas are clinically aggressive neoplasms and may develop de novo from the tubal and/or ovarian surface epithelium. The efficacy of ovarian cancer screening depends on the subtypes of ovarian cancer. Type I ovarian carcinomas are relatively common in Asia, while Type II ovarian carcinomas are relatively common in Europe and the USA. Therefore, annual ovarian cancer screening may improve the prognoses in Asia to a substantially greater degree than in Europe and the USA, as precursors or early-stage Type I ovarian carcinomas can be detected using CA125/TVS in those regions. Furthermore, it is possible to improve the prognosis or induce down-staging of Type II ovarian carcinomas, even in Europe and the USA. The detection of Type II ovarian carcinoma at an early stage remains an unresolved issue. We have likely failed to notice the presence of STICs using CA125/TVS screening alone, as neither method showed positive findings in women with STICs. Biomarkers for the early detection of Type II carcinomas such as STICs are therefore urgently needed [53].

## Figures and Tables

**Table 1:** Characteristics of two types of ovarian carcinoma.

	Type I	Type II
Behavior	Indolent	Aggressive
Genetic instability	Not very unstable	Very unstable
TP 53 mutation	Low	High
*BRCA*1/*BRCA*2 mutation	Low	High
Ki 67 proliferative index	10%–15%	50%–75%
Histological subtype	Endometrioid	High grade serous
	Clear cell	
	Mucinous	
	Low grade serous	
Precursor	Benign cyst	s/o Tubal dysplasia
		(de novo starting)
Discover a precursor	Easy	Difficult
Incidence	Asia > Europe, USA	Europe, USA > Asia
